# Human H7N9 virus induces a more pronounced pro-inflammatory cytokine but an attenuated interferon response in human bronchial epithelial cells when compared with an epidemiologically-linked chicken H7N9 virus

**DOI:** 10.1186/s12985-016-0498-2

**Published:** 2016-03-15

**Authors:** Kelvin K. W. To, Candy C. Y. Lau, Patrick C. Y. Woo, Susanna K. P. Lau, Jasper F. W. Chan, Kwok-Hung Chan, Anna J. X. Zhang, Honglin Chen, Kwok-Yung Yuen

**Affiliations:** Department of Microbiology, The University of Hong Kong, Hong Kong Special Administrative Region, China; State Key Laboratory for Emerging Infectious Diseases, The University of Hong Kong, Hong Kong Special Administrative Region, China; Carol Yu Centre for Infection, The University of Hong Kong, Hong Kong Special Administrative Region, China; Research Centre of Infection and Immunology, The University of Hong Kong, Hong Kong Special Administrative Region, China; Collaborative Innovation Center for Diagnosis and Treatment of Infectious Diseases, Hangzhou, China

**Keywords:** Avian influenza virus, H7N9, Human, Chicken, Cytokine, Chemokine, Interferon, Viral load, Interferon lambda 1, IL-8

## Abstract

**Background:**

Avian influenza virus H7N9 has jumped species barrier, causing sporadic human infections since 2013. We have previously isolated an H7N9 virus from a patient, and an H7N9 virus from a chicken in a live poultry market where the patient visited during the incubation period. These two viruses were genetically highly similar. This study sought to use a human bronchial epithelial cell line model to infer the virulence of these H7N9 viruses in humans.

**Methods:**

Human bronchial epithelial cell line Calu-3 was infected with two H7N9 viruses (human H7N9-HU and chicken H7N9-CK), a human H5N1 virus and a human 2009 pandemic H1N1 virus. The infected cell lysate was collected at different time points post-infection for the determination of the levels of pro-inflammatory cytokines (tumor necrosis factor α [TNF-α] and interleukin 6 [IL-6]), anti-inflammatory cytokines (interleukin 10 [IL-10] and transforming growth factor beta [TGF-β]), chemokines (interleukin 8 [IL-8] and monocyte chemoattractant protein 1 [MCP-1]), and interferons (interferon β [IFN-β] and interferon lambda 1 [IFNL1]). The viral load in the cell lysate was also measured.

**Results:**

Comparison of the human and chicken H7N9 viruses showed that H7N9-HU induced significantly higher levels of TNF-α at 12 h post-infection, and significantly higher levels of IL-8 from 12 to 48 h post-infection than those of H7N9-CK. However, the level of IFNL1 was lower for H7N9-HU than that of H7N9-CK at 48 h post-infection (*P* < 0.001). H7N9-HU had significantly higher viral loads than H7N9-CK at 3 and 6 h post-infection. H5N1 induced significantly higher levels of TNF-α, IL-6, IL-8, IL-10 and MCP-1 than those of H7N9 viruses at 48 h post-infection. Conversely, H1N1 induced lower levels of TNF-α, IL-10, MCP-1, IFNL1 and IFN-β when compared with H7N9 viruses at the same time point.

**Conclusions:**

H7N9-HU induced higher levels of pro-inflammatory IL-6 and IL-8 and exhibited a more rapid viral replication than H7N9-CK. However, the level of antiviral IFNL1 was lower for H7N9-HU than H7N9-CK. Our results suggest that the gained properties in modulating human innate immunity by H7N9-HU transformed it to be a more virulent virus in humans than H7N9-CK.

## Background

The avian influenza H7N9 virus has crossed species barrier, with the first cluster of human infections identified in early 2013 [1–3]. Subsequently, it has spread rapidly across the mainland China, and travel-related cases were identified in Hong Kong, Taiwan, Malaysia and Canada [4–7]. H7N9 virus is the most rapidly spreading zoonotic influenza virus affecting humans, with at least 680 cases in less than 3 years since first identified in humans [8].

H7N9 infection is a highly fatal disease in humans. The case-fatality rate of H7N9 infection in humans is about 40 % [9], which is much higher than that of the 2009 pandemic H1N1 infection [10], but lower than that for H5N1 infection [11]. Unlike most patients with seasonal or pandemic influenza virus infection which present with self-limiting acute upper respiratory illness, the majority of patients with laboratory-confirmed H7N9 infections present with rapidly progressive community-acquired pneumonia, leukopenia, lymphopenia, thrombocytopenia, impaired coagulation profile, deranged liver and renal function, and some patients succumbed with adult respiratory distress syndrome and multiorgan dysfunction [12–15]. These differences in clinical severity and pathology among H7N9, H5N1 and H1N1 may be related to the differential ability of the viruses to induce cytokine and chemokine response. Cytokine and chemokine dysregulation is a hallmark in patients suffering from severe influenza [16–18]. Patients with H7N9 virus infection also had poorer antibody response when compared with those of H5N1 and H1N1 viruses [19]. The suboptimal humoral response induced by H7N9 virus may be related to their internal genes [20].

Epidemiological and phylogenetic analysis suggested that H7N9 virus is primarily transmitted from poultries to humans in live poultry markets (LPM) [2, 21–23]. In our previous study, we have isolated an H7N9 virus from a patient (A/Zhejiang/DTID-ZJU01/2013[H7N9]) (H7N9-HU) and an H7N9 virus from a chicken in an epidemiologically-linked LPM (A/chicken/Zhejiang/DTID-ZJU01/2013[H7N9]) (H7N9-CK) [2]. H7N9-HU and H7N9-CK had high nucleotide similarity. In a subsequent study, we have shown that H7N9-CK could cause lethal infection in mice without prior adaptation, and was associated with high pulmonary levels of pro-inflammatory cytokines [24]. In this study, we used a human bronchial epithelial cell line model to infer the virulence of the human and chicken H7N9 viruses in humans.

## Methods

### Cell and virus isolates

H7N9-HU was isolated from a 64-year-old male patient from Zhejiang who bought live poultry from a LPM within the incubation period, and H7N9-CK was isolated from a chicken from the LPM where that patient bought the live poultry [2]. A/Hong Kong/415742/2009(H1N1) (H1N1) and A/Vietnam/1194/2004(H5N1) (H5N1) were isolated from infected patients [25, 26]. The viruses were propagated in 10-day-old specific-pathogen-free (SPF) chicken embryos at 37 °C for 48 h as we described previously [27]. Aliquots of virus stock were stored at −80 °C until use. Calu-3 cell line (ATCC no. HTB-55) was used for the infection experiments. All experimental protocols followed the standard operating procedures of the approved biosafety level 3 facility as we previously described [25, 26, 28].

### Viral inoculation into Calu-3 cells

Calu-3 cells were seeded onto 96-well tissue culture plates, at 2.5 × 10^4^ cells per well with Dulbecco’s Modified Eagle Medium (DMEM) supplemented with 20 % fetal calf serum (FCS), 100 IU/ml of penicillin, and 100 μg/ml of streptomycin. The culture plates were incubated at 37 °C and 5 % CO_2_ for 48 h prior to the experiment. Cells were inoculated with 3 multiplicity of infection (M.O.I.) of each virus. After 1 h of viral adsorption, the medium was removed and cells were washed twice with medium before further incubation for 3, 6, 12, 24 and 48 h in DMEM containing tosylsulfonyl phenylalanylchloromethyl ketone (TPCK)-treated trypsin (0.5 μg/ml) (Sigma). Cell lysates were collected for cytokine and viral load assays. The experiments were performed in triplicate.

### Determination of the levels of cytokines and chemokines

The levels of cytokines and chemokines were determined by real-time reverse transcription-quantitative polymerase chain reaction (RT-qPCR) as we described previously [25]. RNA extraction was performed using RNeasy Mini Spin Column (QIAgen). The RNA was eluted in 50 μl of RNase-free water and was used as the template for RT-qPCR. Reverse transcription was performed using the oligo(dt) primer with the SuperScript III kit (Invitrogen). RT-qPCR assays for tumor necrosis factor α (TNF-α), interleukin 6 (IL-6), interleukin 8 (IL-8), interleukin 10 (IL-10), transforming growth factor beta (TGF-β), interferon lambda 1 (IFNL1), interferon β (IFN-β), and monocyte chemoattractant protein 1 (MCP-1) were performed as described previously with primers and conditions listed in Table 1, using glyceraldehyde 3-phosphate dehydrogenase (GAPDH) for normalization [25]. cDNA was amplified in a LightCycler 2.0 (Roche) with 20 μl reaction mixtures containing FastStart DNA Master SYBR Green I Mix reagent kit (Roche), 2 μl cDNA, 2 or 4 mM MgCl_2_ and 1 μM primers at 95 °C for 10 min followed by 50 cycles of denaturation, annealing and extension. Melting curve analysis was performed for each primer pair at the end of the reaction to confirm the specificity of the assay.

**Table 1:** Primers and conditions for real-time RT-PCR

Cytokines	Primers					
	Forward	Backward	Mg conc. (mM)	Denaturation	Annealing	Extension
TNF-α	GCCAGAGGGCTGATTAGAGA	CAGCCTCTTCTCCTTCCTGAT	2	95 °C 10s	60 °C 5 s	72 °C 5 s
MCP-1	GCAATCAATGCCCCAGTCA	TGCTGCTGGTGATTCTTCTATAGCT	2	95 °C 10s	55 °C 5 s	72 °C 5 s
IL-6	GGTACATCCTCGACGGCATCT	GTGCCTCTTTGCTGCTTTCAC	2	95 °C 10s	55 °C 5 s	72 °C 5 s
IFN-β	GCCGCATTGACCATCT	CACAGTGACTGTACTCCT	4	95 °C 10s	55 °C 5 s	72 °C 11 s
IFNL1	GAAGCAGTTGCGATTTAGCC	GAAGCTCGCTAGCTCCTGTG	2	95 °C 10s	60 °C 5 s	72 °C 7 s
IL-10	CAAATGAAGGATCAGCTGGACAA	GCATCACCTCCTCCAGGTAAAAC	2	95 °C 10s	55 °C 5 s	72 °C 5 s
TGF-β	CCCAGCATCTGCAAAGCTC	GTCAATGTACAGCTGCCGCA	2	95 °C 10s	55 °C 5 s	72 °C 5 s
IL-8	AGCTGGCCGTGGCTCTCT	CTGACATCTAAGTTCTTTAGCACTCCTT	2	95 °C 10s	55 °C 5 s	72 °C 5 s
GAPDH	ATTCCACCCATGGCAAATTC	CGCTCCTGGAAGATGGTGAT	2	95 °C 10s	55 °C 5 s	72 °C 5 s

### Viral load

To study viral replication kinetics, viral titer from cell lysates collected at 3, 6, 12, 24 and 48 h post-infection were measured by RT-qPCR as described previously [25].

### Statistical analysis

Statistical analysis was performed using GraphPad Prism 6.0. The levels of cytokines and chemokines were expressed as fold-increase from mock-infected cells. The cytokine/chemokine levels and viral loads of different viruses were compared using Student’s t-test when comparing between two viruses, and two-way ANOVA test corrected for multiple comparisons using Tukey post-hoc test when comparing 3 different viruses. A *P* value of <0.05 was considered statistically significant.

## Results

We compared the cytokine and chemokine levels between the human and chicken H7N9 viruses in a human bronchial epithelial cell line Calu-3. At 12 h post-infection, the mean level of TNF-α was significantly higher for H7N9-HU (220-fold) than that of H7N9-CK (55-fold) (*P* = 0.010). The mean level of TNF-α at 24 h post-infection was also higher for H7N9-HU (458-fold) than that of H7N9-CK (224-fold), almost reaching statistical significance (*P* = 0.068). However, at 48 h post-infection, the mean level of TNF-α was similar between H7N9-HU (289-fold) and H7N9-CK (307-fold) (*P* = 0.802). H7N9-HU also induced significantly higher levels of IL-8 from 12–48 h post-infection than those of H7N9-CK. Conversely, the mean level of IFNL1 was significantly lower for H7N9-HU (39-fold) than that of H7N9-CK (55-fold) at 48 h post-infection (*P* = 0.0005). The mean level of MCP-1 was also significantly lower for H7N9-HU than H7N9-CK at 48 h post-infection (*P* = 0.014). There was no significant difference for other cytokines/chemokines.

**Fig. 1:**
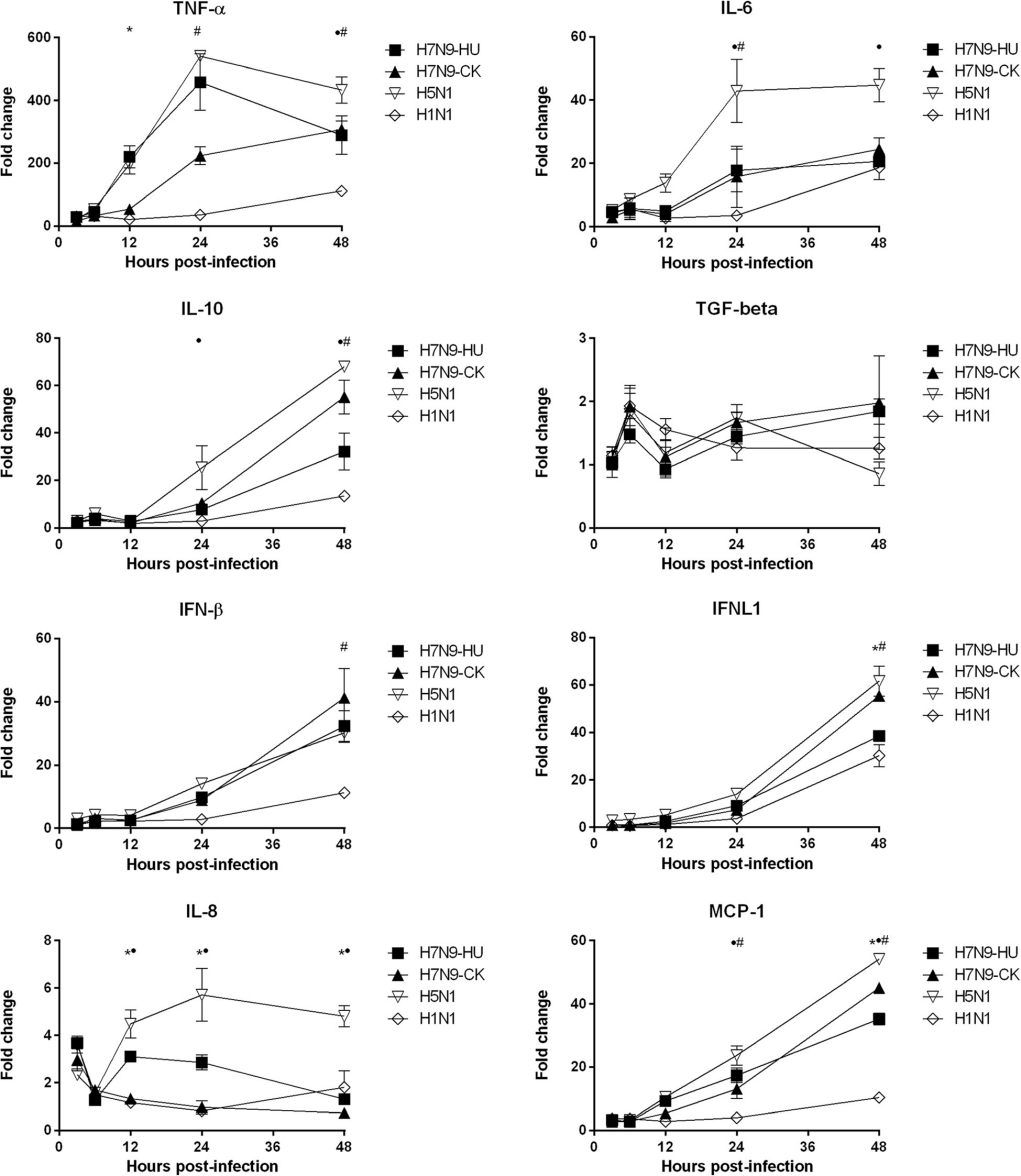
The cytokine/chemokine mRNA levels in Calu-3 cell line infected with A/Zhejiang/DTID-ZJU01/2013(H7N9) (H7N9-HU), A/chicken/Zhejiang/DTID-ZJU01/2013(H7N9) (H7N9-CK), A/Hong Kong/415742/2009(H1N1) (H1N1) and A/Vietnam/1194/2004(H5N1) (H5N1). The experiment was performed in triplicate. Data were shown as mean ± standard error of mean. * indicates *P* < 0.05 when comparing H7N9-HU with H7N9-CK; ^•^ indicates *P* < 0.05 when comparing H5N1 with H7N9-CK or H7N9-HU; # indicates *P* < 0.05 when comparing H1N1 with H7N9-CK or H7N9-HU

Next, we compared the H7N9 viruses with the more virulent H5N1 and the less virulent H1N1 viruses. When compared with H7N9-CK or H7N9-HU, H5N1 induced significantly higher levels of IL-8 at 12 h post-infection; IL-8, IL-6, IL-10 and MCP-1 at 24 h post-infection; and IL-8, IL-6, IL-10, MCP-1 and TNF-α at 48 h post-infection (Fig. 1). Unlike H5N1, H1N1 induced significantly lower levels of IL-6, MCP-1 and TNF-α at 24 h post-infection; and MCP-1, TNF-α, IL-10, IFNL1 and IFN-β at 48 h post-infection when compared with H7N9-CK or H7N9-HU.

**Fig. 2:**
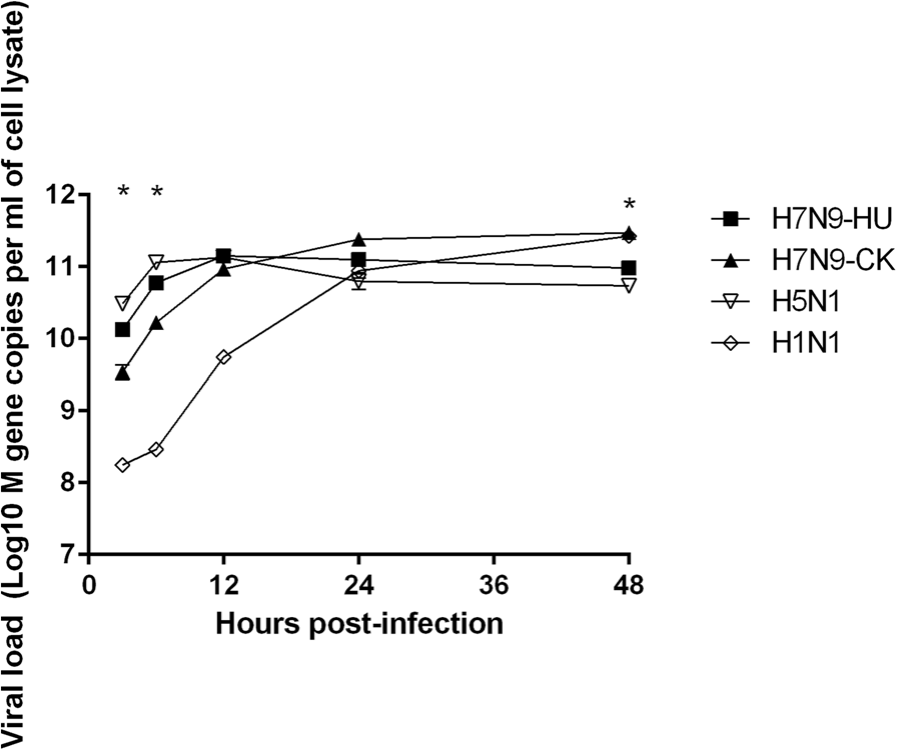
Growth properties of influenza A virus in Calu-3 cell line. The viruses were A/Zhejiang/DTID-ZJU01/2013(H7N9) (H7N9-HU), A/chicken/Zhejiang/1/2013(H7N9) (H7N9-CK), A/Hong Kong/415742/2009(H1N1) (H1N1) and A/Vietnam/1194/2004(H5N1) (H5N1). The experiment was performed in triplicate. Data were shown as mean ± standard error of mean. * indicates *P* < 0.05 when comparing H7N9-HU with H7N9-CK

The viral kinetics were also determined (Fig. 2). At 3 and 6 h post infection, the viral load achieved by H7N9-CK were about 1 log lower than that of H7N9-HU (*P* < 0.005). At 48 h post-infection, the viral load of H7N9-CK was significantly higher than those of H7N9-HU (*P* < 0.001).

## Discussion

Severe influenza is characterized by rapidly progressive pneumonia, acute respiratory distress syndrome and multiorgan failure [11, 12, 14]. An exaggerated innate immune response is observed in severe influenza cases, manifested with elevated levels of pro-inflammatory or anti-inflammatory cytokines and chemokines [15–17, 29, 30]. Although some studies have compared human H7N9 viruses with poultry H7N9 viruses or with other subtypes of human or avian influenza viruses [31–35], there have not been any studies which compared the cytokine/chemokine responses and the replication of H7N9 viruses isolated from human and poultry in epidemiologically-linked markets. In the current study, we compared human and poultry H7N9 viruses that were closely related both epidemiologically and phylogenetically. When compared with the chicken H7N9-CK, the human H7N9-HU induced a stronger TNF-α response at 12 and 24 h post-infection, but a weaker IFNL1 response between 12 and 48 h post-infection. H7N9-HU also replicated more rapidly than H7N9-CK in the first 6 h. H7N9-HU and H7N9-CK are highly similar, with 99.4 % and 99.7 % nucleotide identity in their hemagglutinin and neuraminidase genes, respectively [2]. However, there are two major amino acid differences between the human and chicken H7N9 virus. H7N9-HU has a Gln226Leu substitution (H3 numbering) in the hemagglutinin, which is responsible for an increase binding affinity to the human type α-2,6-linked sialic acid receptor. H7N9-HU also contains an Asp701Asn substitution in PB2, which has been shown to enhance transmission in guinea pigs.

Our cytokine/chemokine findings suggest that H7N9-HU induced a greater proinflammatory response than H7N9-CK. These results corroborate with the results of an *in vivo* model by Zhang et al. [35]. In their study, ferrets infected with human H7N9 strains had greater weight loss, more severe bronchopneumonia, and higher viral loads in the tonsils, trachea and lung than ferrets infected with a chicken H7N9 strain. However, the cytokine/chemokine profiles or other immune profiles of these ferrets were not reported.

We have chosen Calu-3 cell line because bronchial epithelial cell is one of the cell types affected in patients with severe influenza [36]. Calu-3 has been widely used for studies on the pathogenesis of influenza virus and other respiratory viruses [31, 37–39]. McDermott et al. showed that host response induced by high pathogenic avian influenza virus in Calu-3 correlated with that of mouse and macaque models [40].

In this study, we have shown that the level of TNF-α was higher in Calu-3 cells infected with H7N9-HU than that of H7N9-CK. TNF-α is a major pro-inflammatory cytokine. Our results corroborate with those in a study showing higher pulmonary level of TNF-α in mice infected with a human H7N9 virus when compared to that of mice infected with a duck H7N9 virus from 2009 [41]. The level of TNF-α was also increased in H7N9 patients [2], although the level of TNF-α was not associated with the prognosis of patients [17, 42].

The level of IL-8 was also higher for H7N9-HU than that of H7N9-CK. IL-8, or CXCL8, is a chemokine which attracts and activates neutrophils. Previous studies showed that human H7N9 and H5N1 viruses induced higher levels of IL-8 than seasonal H3N2 virus or an H7N9 virus isolated from a shoveler in 2007 [31]. In patients with 2009 pandemic H1N1 virus infection, the levels of IL-8 were also found to be higher in patients with severe disease than those with mild disease [10]. In humans, IL-8 has been associated with acute lung injury [43].

On the contrary, the levels of IFNL1 and MCP-1 were lower in Calu-3 cells infected with H7N9-HU than that of H7N9-CK at 48 h post-infection. IFNL1, also known as IL-29, is an antiviral type III interferon. Administration of IFNL1 can reduce the viral load *in vitro* [44]. In previous studies, avian influenza viruses H5N2 and H9N2 induced robust IFNL1 response in the lung alveolar epithelial A549 cells [45]. MCP-1 is a chemokine mainly responsible for recruiting monocytes. MCP-1 is required for protective response, as treatment of influenza-infected mice with MCP-1 antibody resulted in greater lung damage [46]. However, a higher level of MCP-1 was found in patients who died than those survivors at week 2 post-infection, suggesting that a persistently high level of MCP-1 is associated with poorer prognosis [17].

The viral titers of H7N9-HU were higher than those of H7N9-CK at 3 and 6 h post-infection. This is consistent with another study which showed that the viral titers of the human H7N9 viruses were higher than those of phylogenetically unrelated duck H7N9 virus and the 2009 pandemic H1N1 virus in differentiated normal human bronchial epithelial cells [47]. In another study, a human H7N9 virus was also shown to have better replication in explanted human lung tissue than low-pathogenic avian H7N1 and H7N7 isolated from turkeys [48]. The lower level of IFNL1 and a more rapid viral replication in H7N9-HU-infected cells suggest that H7N9-HU has acquired additional characteristics that enhance the virulence in humans.

Similar to other studies, we have demonstrated that the cytokine/chemokine mRNA expression levels and the viral loads of H7N9, H5N1 and H1N1 virus in Calu-3 cells correlated well with disease severity observed in humans [31]. The avian influenza virus H5N1, which has a case-fatality rate about 53 % [9], induced the highest mRNA expression levels of most tested cytokines/chemokines. On the other hand, the 2009 pandemic H1N1, which has a <1 % case-fatality rate [10], induced the lowest mRNA levels of cytokines/chemokines and the lowest viral load. The avian influenza virus H7N9, which has a case-fatality rate of 40 % [9], induced moderate levels of cytokines/chemokines. H7N9 was also shown to induce a cytokine profile between that of H5N1 and seasonal influenza viruses in primary human macrophages and those in endothelial cells [31, 49].

## Conclusions

In summary, while only few adaptive mutations were observed between H7N9 human and avian viruses, the results of our study suggested that H7N9-HU is more virulent in humans than H7N9-CK. The ability to induce inflammation and the kinetics of viral replication in human cells may be common parameters in predicting the virulence and replication potential of an emerging avian influenza virus in humans.
